# Interval cancers in a population-based screening program for colorectal cancer with gender-specific cut-off levels for fecal immunochemical test

**DOI:** 10.1177/09691413221085218

**Published:** 2022-03-08

**Authors:** Hanna Ribbing Wilén, Deborah Saraste, Johannes Blom

**Affiliations:** Department of Clinical Science and Education Södersjukhuset (KI SÖS), 27106 Karolinska Institutet, Stockholm, Sweden

**Keywords:** Colorectal cancer, screening, fecal immunochemical test, FIT, interval cancer, gender

## Abstract

**Objective:**

To evaluate interval cancers (IC) in the population-based Swedish regional colorectal cancer (CRC) screening program of Stockholm-Gotland, which uses gender-specific cut-off levels for the fecal immunochemical test (FIT).

**Methods:**

All individuals aged 60–69 in Stockholm-Gotland invited to the screening program in October 2015 to September 2017 were followed up 2 years after invitation. Cut-off level for a positive FIT was 40 µg/g in women and 80 µg/g in men. Those with a positive FIT were referred to colonoscopy. Screening-detected CRC (SD-CRC) and IC after negative FIT (FIT-IC) or negative screening colonoscopy (Colonoscopy-IC) were identified in the Swedish colorectal cancer register. The IC rate was calculated as IC/(FIT negatives + negative screening colonoscopies). The IC incidence rate (ICs among negatives per 100,000 person-years) in different sex and age groups was compared to the mean CRC incidence before regional screening implementation. Test sensitivity was defined as SD-CRC/(SD-CRC + FIT-IC).

**Results:**

Approximately 214,400 individuals were invited, and in 3521 screening colonoscopies 257(6.3%) SD-CRCs were detected. During follow-up, 124 FIT-IC and 7 Colonoscopy-IC were diagnosed, yielding an IC rate of 12.6 and 6.0 per 10,000 negatives (*p* = 0.00005) and a test sensitivity of 62% and 75% (*p* = 0.01) in men and women respectively. The IC incidence rate compared to CRC incidence was non-significantly lower in women.

**Conclusion:**

In the population-based screening program of Stockholm-Gotland with a cut-off of 40 µg/g in women and 80 µg/g in men, the test sensitivity was higher and the IC rate was lower in women, which might imply lowering the cut-off level in men. However, the IC incidence rate relative to the CRC incidence was similar in both genders.

## Introduction

Colorectal cancer (CRC) is diagnosed in approximately 1.8 million people worldwide every year, thereby being the second and third most common type of cancer in women and men, respectively, and causing almost 1 million deaths each year.^1^ Fecal immunochemical test (FIT) screening of the average-risk population aims at detecting CRC at an early stage and reducing disease mortality.^2^

The ability of screening to decrease disease mortality in the target population depends on the uptake and the performance of the screening test.^3^ As FIT is more sensitive to advanced neoplasia (CRC and advanced adenoma) than guaiac fecal occult blood test (gFOBT), switching from gFOBT to FIT could decrease the interval cancers (ICs), i.e. the CRCs not detected by the screening program.^4^ The assessment of IC is an important quality measure of the screening program and could be presented as the rate ratio of the background incidence in the population in the period before screening implementation, complemented by the test sensitivity which is the proportion of screening detected CRCs (SD-CRC) of all CRCs.^5,6^

Several studies have indicated that ICs are more common in women.^7^ In the previous population-based gFOBT screening program in the Stockholm-Gotland region of Sweden, an evaluation demonstrated a lower test sensitivity in women as compared to men. Consequently, FIT screening with cut-off levels of 40 µg/g in women and 80 µg/g in men for a positive test was implemented in 2015.^8^ Population-based screening is currently being rolled out at a national level in Sweden and gender-specific screening is also being implemented in Finland. Therefore, an evaluation of the current strategy of the Stockholm-Gotland region is urgent.^9^ We have previously reported the findings and costs of the strategy with gender specific cut-offs.^10^ The aim of the current study was to compare the test sensitivity, the IC rate and the IC incidence rate in relation to the background incidence in men and women in the first screening round.

## Methods

### Study population

In the Stockholm-Gotland region of approximately 2.5 million inhabitants, the biennial FIT colorectal cancer screening program has a coverage of 100% and invites all 60–69-year-olds. There are no exclusions except those from previous screening rounds with high-risk adenomas who are followed up in the polyp surveillance program and not re-invited to screening. The invitations are sent by mail and centrally administrated at the Regional Cancer Center in Stockholm, Sweden. In case of a no-reply a reminder is sent after 8 weeks. The study population consisted of all those invited between October 1^st^ 2015 and September 30^th^ 2017, i.e. one screening round. Since the program is biennial, all participants were followed up until two years from invitation with regard to CRC diagnosis. If two invitations were sent to the same individual during the study period due to, for example, a reminder invitation from a previous screening round and an index invitation for the next screening round, only the first dated invitation was included. The participation rates, as well as colonoscopy findings and screening costs of the program, have been reported previously.^10,11^

### FIT

Since October 1^st^ 2015 the screening program applies gender-specific FIT (OC Sensor, Eiken, Japan) cut-off levels of 40 µg/g for a positive test in women and 80 µg/g in men. Those with a positive test were offered colonoscopy at the nearest participating endoscopy unit. In the case of non-analyzable results, a new test kit was sent. Failure of returning an analyzable result was considered as non-participation. The FIT and colonoscopy results as well as colonoscopy quality parameters were recorded in the screening register.

### CRC definitions

CRC was defined as invasion into the muscularis mucosa layer in the bowel wall. Proximal CRC localization included CRC from caecum to splenic flexure.

Screening detected cancer (SD-CRC) and interval cancers (IC) were classified according to the definitions of the World Endoscopy Organization (WEO): SD-CRC as CRC diagnosed after a positive FIT and positive screening colonoscopy. Non-SD-CRCs were further classified as IC diagnosed before next screening round and after a negative FIT (FIT-IC), after a positive FIT and negative screening colonoscopy (Colonoscopy IC), or after a positive FIT in those not compliant with colonoscopy (IC non-compliant to colonoscopy). CRC in non-participants was not classified as an IC.^5^ All CRCs were identified in the Swedish Colorectal Cancer Register (SCRCR) which has a coverage of 99% and validity of 90%, and matched against the screening register.^12^ The CRCs were assessed according to the TNM classification of malignant tumours, 7th ed. 2010. Tumor stage was determined from pTNM (histopathological TNM) stage in the SCRCR and in case of missing data the preoperative cTNM (preoperative clinical TNM) was used. Stage was further categorized into stage I-II and III-IV respectively.

### Statistics

Participation rate was calculated as the number of individuals with a valid FIT test divided by the number of invited individuals. FIT positivity rate was defined as those with a positive test among FIT participants, and colonoscopy compliance as the rate of individuals with positive FIT who underwent a screening follow-up colonoscopy. The positive predictive value (PPV) was calculated as the number of participants with SD-CRC divided by the number of FIT positives.

Individuals were grouped in gender and age groups (60–64 or 65–69).

The IC rate was calculated as the number of total ICs per 10,000 FIT negatives or FIT positives with negative screening colonoscopy, and calculated for the total screening round and for ICs diagnosed within 0–12 and 13–24 months of screening invitation. The number of screening negatives for 0–12 and 13–24 months respectively was assumed to be half the amount as for the whole screening round of two years. The IC incidence rate was defined as the total number of ICs per 100,000 person-years of follow up regarding CRC diagnosis, i.e. two years for each individual or one year for individuals diagnosed with IC within first year of invitation. Test sensitivity was calculated as the proportion of SD-CRC among those with SD-CRC and FIT-IC. The experienced incidence rate (EIR) was derived from the Swedish Cancer Register of CRCs diagnosed in the decade (1998–2007) before screening was initiated in the Stockholm-Gotland region, and calculated as the mean incidence over 10 years per 100,000 for each age and gender subgroup.^13^ The ratio of IC incidence rate/EIR was calculated for the total IC incidence rate and for ICs occurring 0–12 and 13–24 months from invitation, respectively, and stratified for each age and gender subgroup. The 95% confidence interval (CI) for the rate ratio was calculated with the exact Poisson method.^14^

The same outcome measures were estimated for a cut-off level of 80 µg/g in both genders, assuming SD-CRCs, colonoscopy ICs and CRCs in those non-compliant to colonoscopy in women with FIT 40–79 µg/g all would be classified as FIT-ICs. The ICs were not further specified as diagnosed 0–12 months or 13–24 months after invitation since all the former SD-CRCs with FIT 40–79 µg/g would have been included in the 0–12 months sub-category.

The differences in test sensitivity and IC rate in subgroups, and differences in proportion of CRC characteristics between SD-CRC and FIT-ICs and CRCs in non-participants, were assessed with chi-squared test *p*-values <0.05 were considered statistically significant.

All analyzes were done in R version 4.1.0.^15^

### Ethical permission and consent

The study was approved by the Regional Ethics Board in Stockholm (no. 2019-04850). Informed consent was considered when a participant sent in the test tube. Access to underlying research material can be obtained by email to the corresponding author.

## Results

From October 1^st^ 2015 to September 30^th^ 2017, 214,356 individuals were invited to the screening. The participation rate as well as the compliance with follow-up colonoscopy was higher in women than in men. The overall positivity rate was 2.8% and higher in participants 65–69 years old as compared to 60–64. The PPV for CRC was 6.3% (95% CI 5.6–7.1), and significantly higher in men than in women (Table 1). The total follow-up time was 428,673 person-years with regards to CRC diagnosis.

**Table 1.** Participation and positive predictive values in men and women invited to screening in the Stockholm-Gotland screening program 2015–2017.

Sex, age at invitation	Invited, N	Valid FIT test, N	Participation rate	FIT positive, N (%)	Colonoscopy compliance, N (%)	SD-CRC, N	PPV, % (95% CI)*
Women <65	66,063	46,460	70.3	1201 (2.6)	1043 (86.8)	59	4.9 (3.7–6.1)
Women ≥65	42,794	31,680	74.0	1011 (3.2)	901 (89.1)	63	6.2 (4.7–7.7)
Men <65	66,072	42,159	63.8	1046 (2.5)	910 (87.0)	72	6.9 (5.3–8.4)
Men ≥65	39,427	26,679	67.7	799 (3.0)	667 (83.5)	63	7.9 (6.0–9.8)
All	214,356	146,978	68.6	4057 (2.8)	3521 (86.8)	257	6.3 (5.6–7.1)

FIT = fecal immunochemical test CRC = colorectal cancer. Valid FIT test = number of individuals with an analyzable FIT test Participation rate = number of individuals with a valid FIT/number of individuals invited. FIT positive = FIT ≥ 40 µg/g in women and 80 µg/g in men. SD-CRC = screening detected CRC. PPV = positive predictive value; SD-CRC/FIT positives. CI = confidence interval.

*p = 0.023 for difference in PPV between all men and women; *p* > 0.05 for difference in PPV between all <65 and all ≥65, between men and women <65 and between men and women ≥65.

Among the invited individuals, 257 SD-CRCs, 124 FIT-ICs, 7 colonoscopy ICs, 3 ICs in individuals non-compliant to colonoscopy and 177 CRCs in non-participants were diagnosed within two years of invitation. The overall IC rate was 9.2 (95% CI 7.6–10.7) per 10,000 negatives and significantly higher in men than in women (12.6 vs. 6.0, *p* = 0.00005), as was the IC incidence rate. The test sensitivity was 0.68 (95% CI 0.63–0.72), and significantly higher in women aged 65–69 as compared to men in the same age category (*p* = 0.019) (Table 2).

**Table 2.** FIT results, SD-CRC and IC rates in different age and gender subgroups in the Stockholm-gotland screening program 2015–2017.

Age at invitation and gender	FIT Participants	FIT negatives	FIT positives	Colono-scopies	SD-CRC	FIT-IC	Colonoscopy IC	IC non-compliant to colonoscopy	IC rate (95% CI)*	Test sensitivity (95% CI)**	IC incidence rate (95% CI)
Women <65	46,460	45,259	1201	1043	59	20	2	0	4.8 (2.8–6.7)	0.75 (0.65–0.84)	23.8 (15.7–36.1)
Women ≥65	31,680	30,669	1011	901	63	21	3	1	7.9 (4.8–11.0)	0.75 (0.66–0.84)	39.7 (26.8–58.7)
Men <65	42,159	41,113	1046	910	72	37	2	2	9.8 (6.8–12.8)	0.66 (0.57–0.75)	48.9 (36.0–66.4)
Men ≥65	26,679	25,880	799	667	63	46	0	0	17.4 (12.4–22.4)	0.58 (0.49–0.67)	86.8 (65.1–115.9)
All	146,978	142,921	4057	3521	257	124	7	3	9.2 (7.6–10.7)	0.68 (0.63–0.72)	45.8 (38.7–54.3)

FIT = fecal immunochemical test SD-CRC = screening-detected CRC. IC = interval cancer. FIT-IC = IC after negative FIT. Colonoscopy IC = IC after negative screening colonoscopy. IC rate = number of ICs per 10,000 FIT negatives or FIT positives with negative colonoscopy. Test sensitivity = SD/(SD + FIT IC). IC incidence rate = number of ICs among FIT negatives or FIT positives with negative colonoscopy per 100,000 person-years of follow-up.

*p = 0.000053 for difference in IC rate between men and women. **p = 0.019 for difference in test sensitivity between men and women ≥65.

Of the 134 ICs, 39 (29%) were diagnosed 12 months and 95 (71%) 13–24 months from invitation, and consequently the IC rate was higher the second year after invitation (Table 3). The overall ratio of IC incidence rate/EIR ranged from 0.30 to 0.44 in women and men, and from 0.29 to 0.57 and 0.83 to 1.18 0–12 and 13–24 months after invitation, respectively. The rate ratios were slightly lower in women than in men, but not statistically significant (Table 3).

**Table 3.** IC incidence rate in relation to experienced incident rate (EIR) and by year from invitation in the Stockholm-gotland screening program 2015–2017.

					IC 0–12 months from invitation				IC 13–24 months from invitation			
Age at invitation and gender	Total IC, N	IC incidence rate (95% CI)	EIR	IC incidence rate/EIR (95% CI)	Observed ICs	IC rate (95% CI)	IC incidence rate (95% CI)	IC incidence rate/EIR (95% CI)	Observed ICs	IC rate (95% CI)	IC incidence rate (95% CI)	IC incidence rate/EIR (95% CI)
Women <65	22	23.8 (15.7–36.1)	78.3	0.30 (0.18–0.49)	7	3.02 (1.21–6.22)	30.2 (12.1–62.2)	0.39 (0.15–0.84)	15	6.47 (3.62–10.7)	64.7 (36.2–106.6)	0.83 (0.44–1.45)
Women ≥65	25	39.7 (26.8–58.7)	131.8	0.30 (0.19–0.46)	6	3.80 (1.39–8.26)	38.0 (13.9–82.6)	0.29 (0.10–0.64)	19	12.0 (7.24–18.8)	120.2 (72.4–187.7)	0.91 (0.53–1.48)
Men <65	41	48.9 (36.0–66.4)	123.8	0.39 (0.27–0.57)	11	5.23 (2.61–9.35)	52.3 (26.1–93.5)	0.42 (0.21–0.78)	30	14.3 (9.62–20.4)	142.6 (96.2–203.5)	1.15 (0.74–1.72)
Men ≥65	46	86.8 (65.1–115.9)	198.3	0.44 (0.31–0.61)	15	11.3 (6.31–18.6)	112.7 (63.1–185.9)	0.57 (0.31–0.96)	31	23.3 (15.8–33.1)	232.9 (158.3–330.6)	1.18 (0.78–1.72)

CRC = colorectal cancer. IC = interval CRC. EIR = experienced incidence rate; mean value in Stockholm-Gotland region for the years 1998–2007 in different age and gender groups per 100,000. IC incidence rate = number of ICs per FIT negatives or FIT positives with negative colonoscopy per 100,000 person-years. IC rate = number of ICs per 10,000 FIT negatives or FIT positives with negative colonoscopy. CI = confidence Interval.

In Table 4, the outcome measures were estimated if the cut off-level had been 80 µg/g in both genders. The overall test sensitivity was 0.59; 0.56 and 0.62 in women and men respectively (*p* = 0.259). The IC rate was 9.75 and 12.7 in men and women respectively (*p* = 0.106). The corresponding IC incidence rate/EIR would range between 0.39 to 0.54 in men and women of both age categories.

**Table 4.** Estimations of test sensitivity, IC rate and IC incidence in relation to experienced incident rate (EIR) of CRC with cut-offs 80 µg/g in both genders.

Age at invitation and gender	FIT positive	FIT negative	Colonoscopies	SD-CRC	FIT- IC	Colonoscopy IC	IC non-compliant to colonoscopy	Test sensitivity (95% CI)*	IC rate (95% CI)**	EIR	IC incidence rate/EIR (95% CI)
Women <65	688	45,772	609	42	38	1	0	0.52 (0.42–0.63)	8.4 (5.8–11.1)	78.3	0.54 (0.36–0.80)
Women ≥65	542	31,138	491	51	36	1	0	0.59 (0.48–0.69)	11.7 (7.9–15.5)	131.8	0.44 (0.30–0.64)
Men <65	1046	41,113	910	72	37	2	2	0.66 (0.57–0.75)	9.8 (6.8–12.8)	123.8	0.39 (0.27–0.57)
Men ≥65	799	25,880	667	63	46	0	0	0.58 (0.49–0.67)	17.4 (12.4–22.4)	198.3	0.44 (0.31–0.61)

SD-CRC = screening-detected CRC. IC = interval cancer. FIT-IC = IC after negative FIT. Colonoscopy IC = IC after negative screening colonoscopy. Test sensitivity = SD/(SD + FIT IC). IC rate = number of ICs per 10,000 FIT negatives or FIT positives with negative colonoscopy. EIR = experienced incidence rate; mean value in Stockholm-Gotland region for the years 1998–2007 in different age and gender groups per 100,000. IC incidence rate = number of ICs per FIT negatives or FIT positives with negative colonoscopy per 100,000 person-years.

*p = 0.259 for difference in test sensitivity between men and women. **p = 0.106 for difference in IC rate between men and women.

In Table 5, the test sensitivities in men and women, for proximal and distal CRC localization, and in different age categories are specified. Test sensitivity was significantly higher in women than in men (0.75 vs. 0.62) and for distal than for proximal CRCs (0.75 vs. 0.52).

**Table 5.** Test sensitivity for men and women, in different age categories and for proximal and distal CRC localization in the Stockholm-Gotland screening program 2015–2017.

	SD-CRC	FIT-IC	Test sensitivity, %	Difference in sensitivity (p-value)
Age at invitation				
60–64 years	131	57	69.7	4.4 (0.42)
65–69 years	126	67	65.3	
Sex				
Men	135	83	61.9	12.9 (0.011)
Women	122	41	74.8	
CRC localization^a^				
Proximal	65	60	52.0	23.3 (0.0000089)
Distal	192	63	75.3	

FIT = fecal immunochemical test CRC = colorectal cancer. SD-CRC = screening-detected CRC. IC = interval cancer. FIT-IC = IC after a negative FIT. Test sensitivity = SD-CRC/(SD-CRC + FIT IC).

^a^One FIT-IC with unknown localization was excluded.

In Table 6, age, gender, CRC localization and stage distribution were compared regarding the different modes of CRC detection (SD-CRC, FIT-IC and CRC in non-participants). The proportion of women was higher in SD-CRC versus FIT-IC (*p* = 0.011). Stage I and stage II CRC was significantly more common in SD-CRC (55.3%) than for the other modes of CRC detection. The proportion of proximal CRC was significantly lower in SD-CRC (25.3%) than in FIT-IC (48.4%) and CRCs in non-participants (37.9%).

**Table 6.** Comparisons of SD-CRCs, IC and CRCs in non-participants regarding age, gender, CRC localization and stage.

	SD-CRC, N (% of SD-CRC)	FIT-IC, N (% of FIT-IC)	Colonoscopy IC, N (% of Colonoscopy IC)	CRC in non-participants, N (% of CRC in non-participants)
Age				
<65	131 (51.0)	57 (46.0)	4 (57.1)	96 (54.2)
≥65	126 (49.0)	67 (54.0)	3 (42.9)	81 (45.8)
Gender*				
Women	122 (47.5)	41 (33.1)	5 (71.4)	68 (38.4)
Men	135 (52.5)	83 (66.9)	2 (28.6)	109 (61.6)
CRC localization**				
Proximal	65 (25.3)	60 (48.4)	2 (28.6)	67 (37.9)
Distal	192 (74.7)	63 (50.8)	5 (71.4)	110 (62.1)
No localization	0	1 (0.8)	0	0
CRC stage***				
I&II	142 (55.3)	47 (37.9)	5 (71.4)	63 (35.6)
III&IV	89 (34.6)	58 (46.8)	1 (14.3)	91 (51.4)
No stage	26 (10.1)	19 (15.3)	1 (14.3)	23 (13.0)
Total	257	124	7	177

CRC = colorectal cancer. FIT = fecal immunochemical test SD-CRC = screening-detected CRC. IC = interval CRC. FIT-IC = IC after a negative screening FIT. Proximal CRC = CRC located in caecum to splenic flexure. CRC stage I&II = CRC confined to bowel wall. CRC stage III&IV = CRC with regional lymph node metastases or distant metastases.

*p = 0.011 for gender in SD-CRC vs FIT-IC; *p* = 0.077 for gender in SD-CRC vs CRC in non-participants.

**p = 0.0000089 for CRC localization in SD-CRC vs FIT-IC (the CRC with unknown localization was excluded from analysis); *p* = 0.0072 for CRC localization in SD-CRC vs CRC in non-participants.

***p = 0.0061 for CRC stage in SD-CRC vs FIT IC; *p* = 0.00011 for CRC stage in SD-CRC vs CRC in non-participants (CRC with unknown stage excluded from analysis).

Of all 568 CRCs, 196 (34.5%) were proximally located, and proximal localization was more common in women than in men (42% vs. 29%, *p* = 0.0030). One CRC with unknown localization was excluded from analysis.

## Discussion

This is the first assessment of interval cancers in a screening program that applies gender-specific cut-off levels for a positive test In the Stockholm-Gotland screening program in Sweden, a cut-off level of 40 µg/g in women and 80 µg/g in men yielded a higher test sensitivity in women as compared to men, and a higher IC rate in men than in women. However, when taking the background CRC incidence into account, the gender differences in screening program performance were less obvious. The evaluation was imperative as FIT-based screening is being implemented nationally in Sweden and gender-based screening is being rolled out or called for in other European countries.^9,16^

The IC incidence rate in the present study was 46 per 100 000 person-years. This is much higher than that of a recent meta-analysis by Wieten et al. but most of the included studies were in younger populations, applied a cut-off 20 µg/g or lower, and involved multiple screening rounds.^17^ The IC incidence rate decreases after the first screening round because with multiple rounds there is an increase in SD-CRCs as well as in screening negatives and years of follow-up.^17,18^ It is likely that over repeated FIT screening rounds the IC incidence would be lower, although some individuals in the study population had participated in a gFOBT program and were not screening-naive.

Moreover, the IC rate (and consequently the IC incidence rate) was significantly higher in men than in women with the gender-specific screening strategy and was estimated to be more similar with a cut-off level of 80 µg/g in both genders. The higher IC rate in men is explained by the higher cut-off level yielding more ICs in men, but also reflects the higher CRC incidence in men. Because the CRC incidence is lower in women a lower IC rate is expected, although this could be counterbalanced by the higher participation rate in women. Indeed, the IC incidence rate was similar in men and women in the above-cited meta-analysis by Wieten et al.^17^ However, the higher IC rate in men could indicate that the difference in cut-off level between men and women has become too large and disadvantageous to men.

The IC rate is a valuable indicator of the performance of a screening program, since it measures the number of missed CRCs in relation to the number of screening negatives.^5^ However, it does not account for the potential bias that healthier subjects with a lower risk of CRC could be more likely to participate in screening.^19–21^ Hence, the overall IC rate could be underestimated because of a certain degree of selection bias when compared to the background incidence. In the present study, we compared the IC incidence rate relative to the EIR in age- and gender-specific subgroups to evaluate gender-specific screening and found that the IC incidence rate in relation to background incidence was similar in men and women - although there was a non-significant tendency toward a lower rate ratio in women - concluding that in one screening round the program misses CRCs in men and women in the same proportions as they are expected to appear in the population. However, future studies are needed to address this over multiple screening rounds. In programs with gender-equal cut-off levels, the IC incidence relative to the background incidence has been higher in women than in men: in an Italian study of screening programs using cut-off level 20 µg/g in both genders, the proportional incidence of IC (calculated from the expected number of CRCs had screening not been initiated and the observed number of ICs) was significantly higher in women than in men (21% vs. 15%).^22^ In a study from the Veneto region in Italy over multiple screening rounds with FIT cut-off level 20 µg/g in both genders, the sensitivity of the program was calculated as 1 minus the proportional incidence of IC and was significantly higher in men (89%) than in women (82%).^18^

In the present study, the IC incidence/EIR was well below one for the total follow-up period of two years. This is expected since screening detects CRCs and because there is probably a selection of healthier individuals participating in screening who could have a lower CRC risk than the general population. In all age and gender groups the IC incidence rate/EIR was higher in the second year as compared to the first year after screening invitation because most ICs were diagnosed in the second year. However, in men in the second year after screening the IC incidence/EIR exceeded one, which seems counterintuitive since it implies that there would be more CRCs in the second year after screening than there would have been without screening. Likewise, in the previous gFOBT program in 2011 the IC rate/EIR exceeded 100%.^8^ On the other hand, in the present study the numbers were few and the 95% CIs were wide, so the estimate should be interpreted with caution. Moreover, there is a difference between the EIR and the IC incidence rate in that the screening cohort is followed for two years, and hence the screening cohort is older in the second year after screening (61–65 and 66–70 years respectively), since age is measured at screening invitation. The EIR of 61–65 and 66–70 year olds is probably higher than for 60–64 and 65–69 year olds, and therefore the IC incidence rate/EIR is probably overestimated the second year after invitation.

In FIT screening, a meta-analysis including 31 studies has demonstrated a test sensitivity for CRC between 71% and 91% depending on the cut-off level.^23^ The test sensitivity is also an important quality measure of a screening program but takes only the rate of screening-detected versus the total number of CRCs into account and is thus dependent on the participation rate. It Is likely that the test sensitivity decreases with repeated screening rounds as asymptomatic and preclinical CRCs are detected in the first round and the number of SD-CRCs declines as precursors to CRC are removed among those screened.^24^ The test sensitivity in the present study (68%) represents that of a population of both screening-naïve 60-years-olds and those previously screened with gFOBT, and was higher than in the Scottish screening program (50%) with cut-off 80 µg/g in both men and women, as well as in the previous gFOBT program in the Stockholm-Gotland region (42%).^8,25^

The gender-specific screening strategy in Stockholm-Gotland rendered a significantly higher test sensitivity in women than in men and was estimated to be equalized had the cut-off levels been 80 µg/g in both genders. However, the participation rate was significantly higher in women than in men, which might have contributed to the gender difference in test sensitivity. Moreover, in the previous gFOBT program of Stockholm-Gotland the sensitivity was lower in women than in men, which also could add to the gender difference in test sensitivity seen in the FIT program.^8^ Most of the CRCs in the gFOBT program would become symptomatic and be diagnosed outside the program within two years, thus classified as ICs of the gFOBT program, but there is a possibility that a small proportion of CRCs remained asymptomatic and undiagnosed for two years, thus becoming SD-CRC or ICs (or CRCs in non-participants) in the subsequent FIT screening round. This could yield a higher test sensitivity for FIT in women. On the other hand, the switch from gFOBT to FIT increased the participation rate more in men than in women, which might render a proportionally larger increase in test sensitivity in men in the FIT as compared to the gFOBT program.^11^ For advanced adenomas (AAs), test sensitivity for one round of gFOBT is approximately 10% and significantly higher in men than in women, so most AAs would have remained undiagnosed in both men and women in the previous gFOBT round.^26,27^ In the subsequent FIT round, some of these AAs would be diagnosed as AA, since FIT has a higher sensitivity than gFOBT for AA, or have progressed to CRC and thus contributed to a slightly higher test sensitivity in women.

In the above-mentioned Scottish study, there were few CRCs and the difference in test sensitivity between men and women was not statistically significant.^25^ In the first round of the Dutch screening program, with cut-off levels 15 µg/g in the early and 47 µg/g in the later study period, test sensitivity was 87% in men and 83% in women (*p* < 0.001).^28^

FIT sensitivity is lower in proximal versus distal advanced neoplasia possibly because these lesions are less prone to bleeding due to the flat appearance and due to the degradation of hemoglobin during colonic transit, and in some studies FIT sensitivity for CRC is also lower for proximal versus distal localization.^26,29,30^ The reasons for test sensitivity to be lower in women than in men in gender-uniform FIT screening could be the higher rate of proximal CRCs in women.^31^ In our study, test sensitivity was higher in distal versus proximal CRC (along with a higher rate of proximal CRC in FIT-ICs than in SD-CRCs). Moreover, proximal localization was more common in women than in men. In the screening program in the Italian region Emilia-Romagna, a similar result was seen with a higher proportional incidence of IC in women and for proximal CRCs,^20^ and over repeated screening rounds in the Veneto region in Italy, the proportional incidence of proximal IC was higher as compared to that of the distal.^32^

ICs could arise from a false negative FIT, from a missed lesion, or from incomplete resection of a precursor at colonoscopy. Most of the ICs in our study were FIT-ICs detected 13–24 months from invitation (71%). This raises the question as to whether the ICs were missed by FIT or were fast-growing de novo tumors that appeared after screening. A previous study of the histopathology of FIT-ICs revealed a higher rate of a more aggressive phenotype compared to SD-CRCs, indicating that some FIT-ICs might in fact be faster growing new tumors with correctly negative FIT.^33^ However, in the present study the proportion of stage III-IV CRC was high in the FIT-ICs which may indicate that these FIT-ICs were truly missed.

As the aim of CRC screening is to detect CRC at an early curable stage, evident in the high proportion of early-stage SD-CRC as compared to that of non-participants, future studies will address the disease-specific mortality of the program.

This study has several limitations. First, the follow-up period was only with regard to the diagnosis of CRC and not censored for migration or death. There is the possibility that individuals migrated to other countries or died from causes other than CRC within two years of invitation, thereby underestimating the number of ICs. However, this number of individuals is likely very small.

Secondly, since screening was initiated in 2008 in the region, the mean EIR is based on incidence numbers from a long time ago and the background CRC incidence could have changed since that period. However, from 2009 to 2019 the incidence of CRC in 60–69 years olds in Sweden has been stable at around 150 and 110 per 100,000 in men and women, respectively, except for a peak incidence in women in 2015–2017.^34^ Additionally, more recent incidence numbers from other regions may not be representative as a background incidence in Stockholm-Gotland.

Thirdly, the estimated ICs of a screening strategy with a cut-off level of 80 µg/g in both genders were based on the assumption that all CRCs in women with FIT 40–70 µg/g would be classified as FIT-ICs. However, there is a possibility of a small proportion of these CRCs remaining undiagnosed for two years until the next screening round, thereby overestimating the IC rate and the IC incidence rate/EIR in women with this strategy for the present screening round.

Moreover, we defined age as ‘age at invitation’ throughout the study and not ‘age at diagnosis’ because most invitees did not have a CRC diagnosis. The EIR of 61–65 and 66–70 year olds was not available. As a result, the IC incidence rate/EIR in the second year after screening was based on the ages at invitation and not the ages at diagnosis, hence the rate ratio is probably overestimated in the second year, as discussed above.

The strength of this study is that it is the first evaluation of ICs in a populations-based invitational screening program with gender-specific cut-off levels, where all CRCs were identified in the SCRCR which has a coverage of 99%.^12^

In conclusion, the first screening round of the Stockholm-Gotland program with gender-specific cut off-levels for a positive FIT demonstrated a higher test sensitivity in women than in men, and a higher IC rate in men than in women. However, the gender-differences were non-significant when the incidence rate of IC was compared to the background incidence stratified by different age- and gender groups. If larger studies confirm the tendency of lower IC incidence relative to the background incidence in women, men are disadvantaged with this screening strategy and an adjustment of cut-off levels should be considered.
